# Appropriateness and timeliness of care-seeking for complications of pregnancy and childbirth in rural Ethiopia: a case study of the Maternal and Newborn Health in Ethiopia Partnership

**DOI:** 10.1186/s41043-017-0120-2

**Published:** 2017-12-21

**Authors:** Lynn M. Sibley, Yared Amare, Solomon Tesfaye Abebe, Mulusew Lijalem Belew, Kemeredin Shiffra, Danika Barry

**Affiliations:** 1 Nell Hodgson Woodruff School of Nursing and Rollins School of Public Health, Emory University, Atlanta, GA 30322 USA; 2 Consultancy for Social Development, Addis Ababa, Ethiopia; 3 Addis Ababa, Ethiopia; 4 Amhara Regional Health Bureau, Bahirdar, Ethiopia; 5 Oromia Regional Health Bureau, Addis Ababa, Ethiopia; 6 Harvard Medical School, Cambridge, MA 30138 USA

**Keywords:** Illness recognition, Care-seeking, Maternal and newborn complications, Community-oriented interventions

## Abstract

**Background:**

In 2014, USAID and University Research Co., LLC, initiated a new project under the broader Translating Research into Action portfolio of projects. This new project was entitled *Systematic Documentation of Illness Recognition and Appropriate Care Seeking for Maternal and Newborn Complications*. This project used a common protocol involving descriptive mixed-methods case studies of community projects in six low- and middle-income countries, including Ethiopia. In this paper, we present the Maternal and Newborn Health in Ethiopia Partnership (MaNHEP) case study.

**Methods:**

Methods included secondary analysis of data from MaNHEP’s 2010 baseline and 2012 end line surveys, health program inventory and facility mapping to contextualize care-seeking, and illness narratives to identify factors influencing illness recognition and care-seeking. Analyses used descriptive statistics, bivariate tests, multivariate logistic regression, and thematic content analysis.

**Results:**

Maternal illness awareness increased between 2010 and 2012 for major obstetric complications. In 2012, 45% of women who experienced a major complication sought biomedical care. Factors associated with care-seeking were MaNHEP CMNH Family Meetings, health facility birth, birth with a skilled provider, or health extension worker. Between 2012 and 2014, the Ministry of Health introduced nationwide initiatives including performance review, ambulance service, increased posting of midwives, pregnant women’s conferences, user-friendly services, and maternal death surveillance. By 2014, most facilities were able to provide emergency obstetric and newborn care. Yet in 2014, biomedical care-seeking for perceived maternal illness occurred more often compared with care-seeking for newborn illness—a difference notable in cases in which the mother or newborn died. Most families sought care within 1 day of illness recognition. Facilitating factors were health extension worker advice and ability to refer upward, and health facility proximity; impeding factors were time of day, weather, road conditions, distance, poor cell phone connectivity (to call for an ambulance), lack of transportation or money for transport, perceived spiritual or physical vulnerability of the mother and newborn and associated culturally determined postnatal restrictions on the mother or newborn’s movement outside of the home, and preference for traditional care. Some families sought care despite disrespectful, poor quality care.

**Conclusions:**

Improvements in illness recognition and care-seeking observed during MaNHEP have been reinforced since that time and appear to be successful. There is still need for a concerted effort focusing on reducing identified barriers, improve quality of care and provider counseling, and contextualize messaging behavior change communications and provider counseling.

## Background

In 2014, the US Agency for International Development Translating Research into Action Project (USAID/TRAction) launched a “Systematic Documentation of Illness Recognition and Appropriate Care Seeking for Maternal and Newborn Complications” through the University Research Co., LLC, (URC) [1].The review emphasized a descriptive mixed-methods case study approach [2]. It was motivated by the lack of evidence as to how women and families identify maternal and newborn complications, factors underlying the decision to seek care, and the role of cultural beliefs as drivers of perceptions and behavior at family and community levels—evidence that is necessary for informing the design of interventions to increase the use of health services and decrease maternal and newborn mortality in low- and middle-income countries [1].

The review included one current or recently completed community-oriented project from each of the following countries: Ethiopia, India, Indonesia, Nigeria, Tanzania, and Uganda. The review was framed by the delay model [3] and included maternal and health program inventory and health facility mapping to contextualize the setting in which care-seeking occurs, as well as illness narrative to examine facilitators and barriers to recognition and appropriate care-seeking. Some country teams also conducted secondary analysis of project data as well as baseline and end line surveys to augment description. The Maternal and Newborn Health in Ethiopia Partnership (MaNHEP) project was selected from Ethiopia [4].

### Ethiopia and the MaNHEP project

When MaNHEP launched in 2010, Ethiopia had great needs in the area of maternal and newborn health. The reported maternal mortality ratios ranged from 350 to 676 deaths per 100,000 live births, varying by the source [5, 6]. While the country had made considerable progress in reducing child mortality, there was essentially no change in the neonatal mortality rate between 2005 and 2010 (from 39 to 37 deaths per 1000 live births) [6, 7]. A combination of factors contributed to the high levels of maternal and newborn mortality. Ethiopia’s population is predominantly rural (85%). At the time, coverage was very low for skilled antenatal, delivery, and postnatal care (50, 10, and 7%, respectively). Most women gave birth at home (90%) attended by family members or traditional birth attendants, and most deaths occurred at home around the time of birth [6, 7]. Ethiopia’s flagship Health Extension Program, intended to extend the reach of preventive health services to communities, was envisaged but not fully in place, especially with regard to extending the reach of maternal, newborn, and child health services [4].

MaNHEP was a learning project to develop and position for scale-up a community-oriented model of maternal and newborn health focusing on the time around birth when the risk of maternal and newborn death is greatest [4]. It aimed to strengthen the Government of Ethiopia’s flagship Health Extension Program to more effectively extend the reach of maternal and newborn health services. MaNHEP operated with sponsorship of the Federal Ministry of Health. The project was implemented by Emory University in collaboration with the Amhara and Oromia Regional Health Bureaus, John Snow Research and Training Inc., University Research Co., LLC, and Addis Ababa University. The objectives were to build confidence and competence of frontline health workers to provide targeted care around the time of birth, increase demand for such care and improve self-care practices of women and families, and develop model *woredas* (districts) capable of continuously improving maternal and newborn health service delivery to meet the needs of childbearing families.

There were four interventions to achieve these objectives. One was refresher clinical training in safe, clean delivery, prevention of postpartum hemorrhage, newborn resuscitation, and postnatal care for health extension workers. Another involved community maternal and newborn health (CMNH) family meetings for groups of women in their second and third trimester of pregnancy along with their family caregivers—those who would be present at birth. The meetings, led by project-trained community health volunteers and supported by government health extension workers, focused on birth preparedness and complication readiness for maternal and newborn complications; care during labor, birth, and the postnatal period; and prevention of postpartum hemorrhage and simple newborn resuscitation. A third intervention involved training in continuous quality improvement methods and coaching for teams comprised of community stakeholders and health service managers. The final intervention was behavior change communications focusing on the value of healthy mothers and newborns and the importance of maternal and newborn health care. Communications were delivered through a variety of locally appropriate channels such as community meetings, radio spots, mobile video drama, and poetry contests [4].

MaNHEP interventions were associated with significant improvements in the completeness of CMNH care provided by project-trained health extension workers and community health volunteers (calculated as the average proportion of 17 CMNH care elements provided at the last birth they attended across individuals surveyed) in their demonstrated capacity (calculated as the average proportion of 17 CMNH care elements demonstrated against skills checklists using observation of caregiving scenarios across individuals surveyed) and confidence to provide care (calculated as the average level of confidence in ability to provide antenatal, birth, postpartum, and newborn care, respectively, using a 10-point Likert-type scale, across individuals surveyed) and in their sense of being part of a care team (calculated as the average proportion of yes responses across individuals surveyed) [4].

There were also significant improvements in women’s awareness and level of trust in the ability of these team members to provide care, in the completeness of care women received, and in their use of skilled providers or health extension worker for antenatal and postnatal care [4]. A small shift occurred toward use of skilled providers and health facilities for birth. Successful local solutions for pregnancy identification, antenatal care registration, labor-birth notification, and postnatal follow-up were adopted across 51 project *kebeles* (communities) [4].

Finally, the project team identified, enrolled, and followed a cohort of approximately 9500 pregnant women residing in the project communities from March 1, 2011, through February 28, 2012. The team conducted verbal autopsy in all cases of perinatal death, which included data on the dates of birth and death for 175 deaths among this cohort. A statistical control process G-chart was used to produce an estimate of the number of days between deaths. A significant shift in the mean number of days between deaths occurred in December 2012. There was a significant increase in the number of days between the 175 newborn deaths, beginning about 9 months after the rollout of CMNH family meetings and quality improvement activities. By 1 year, the interval between deaths began to exceed the upper control limit, indicating that there was some special cause for the variation, not normal monthly fluctuations in the frequency of deaths [4]. Other community-oriented projects such as MaNHEP have also been associated with improvements in newborn health outcomes [8, 9]. MaNHEP was selected for a case study based on these promising results.

### The MaNHEP case study

Emory University, non-governmental organization Consultancy for Social Development, and the Amhara and Oromia regional health bureaus implemented the MaNHEP case study, conducted over a 9-month period. The study aimed to gain a more nuanced understanding of factors that facilitate or impede illness recognition, care-seeking decisions, and care-seeking behaviors for pregnancy-related complications in context.

In this paper, we focus on a specific set of questions:

- To what extent was MaNHEP associated with improvements in care-seeking for pregnancy-related complication between the 2010 baseline and 2012 end line?
- What was the context of care-seeking in the former MaNHEP project area in 2014? In what ways did this context influence care-seeking pathways, timing, and experience?
- Were there differences in care-seeking pathways and timing for maternal compared with newborn illness events in 2014? If so, how can these be explained?

We discuss advantages and challenges of using a descriptive case study approach to understand factors that facilitate or impede illness recognition and appropriate care-seeking, similarities and differences in such factors identified in this study compared with others, and insights gained by comparing maternal and newborn illness recognition and appropriate care-seeking and implications for programming and research.

## Methods

### Case study sites

The MaNHEP project area and site of this case study covered six *woredas* (districts) comprised of 51 *kebeles* (communities) located in Amhara and Oromia regions of Ethiopia [4]. The *woredas*, predominantly rural agricultural, have a combined estimated population of 350,000. Each has an urban center and about six health centers, each of which oversees five to six health posts. A hospital capable of providing comprehensive emergency obstetric and neonatal care was within 2 h of the project area communities. Two female health extension workers who are responsible for health promotion and some basic curative services staff the health posts. In 2011/2012, the Ministry of Health introduced the Health Development Army to extend the reach of the Health Extension Program. These community health volunteers, many of whom were former MaNHEP-trained volunteers, work under the health extension workers and operate at a ratio of one worker to five households [10, 11].

### Secondary analysis of MaNHEP’s baseline and end line data

We conducted a secondary analysis of the MaNHEP 2010 baseline and 2012 end line survey data [12–14] to assess changes in awareness of pregnancy-related complications, care-seeking among women who experienced a complication, and factors that may be associated with care-seeking. The surveys involved systematic random samples of 1027 and 1019 women of reproductive age who gave birth within 1 year before the surveys, respectively. The survey sampling procedure was as follows: 10 *kebeles* were randomly selected from each study *woreda* for a total of 60 *kebeles* in the baseline survey and 51 *kebeles* in the end line survey. In each of these *kebeles*, two data collection teams started at a locally defined central point*.* They proceeded in opposite directions from this center, first calling on the third household and subsequently calling on every other household. If a household did not contain anyone eligible for the survey, the team moved to the household next door and resumed their sampling system after they had identified an eligible household. In households, in which there was more than one eligible person, the teams asked for one to volunteer or, in the event that all volunteered, randomly chose one person to interview using a structured questionnaire.

We obtained a subset of clean data contained in a database housed at Emory University. Variables of interest were women’s awareness of danger signs and experience of complications in the most recent birth, care-seeking for complications, type of individual facilitating referral, and factors possibly associated with care-seeking (i.e., age, parity, residence, education, land ownership, antenatal care attendance, MaNHEP community maternal and newborn health family meeting attendance, travel time to facility, type of birth attendant, place of birth, and prior infant death).

The baseline and end line survey questionnaires contained questions with pre-coded response options for awareness of common symptoms of complications. The options included anemia, severe headache, blurred vision, high blood pressure, swelling of hands or face, fits, vaginal bleeding, high fever, foul smelling vaginal discharge, severe abdominal pain, labor lasting longer than 12 h, ruptured uterus, mal-presentation (any part of the baby other than the head is seen in the birth passage, like buttocks, hand, foot, or cord), decreased/absent fetal movement, and cord around baby’s neck. To elicit women’s awareness of danger signs, the women were asked a single open-ended question, “Can you tell me what are all of problems that can happen during pregnancy, labor, and after delivery that require immediate attention from a trained health care worker or health facility?” In the end line survey, to elicit women’s experience of complications, they were additionally asked, “Did you or your baby experience any complications while you were pregnant, during labor/delivery or after the baby was born?” If yes, “What complications did you or your baby experience?” And, “Were you referred to a higher facility (health center or hospital) for this complication?” “Who helped facilitate your referral?” [13, 14].

The women mentioned a number of symptoms. To permit analysis, we categorized these responses into conditions associated with maternal illness and death [12–14]: excessive bleeding (e.g., any bleeding during pregnancy, retained placenta, excessive bleeding after birth), obstructed labor (e.g., labor > 12 h, mal-presentation, and ruptured uterus), symptoms suggesting pre-eclampsia or eclampsia (e.g., severe headache, blurred vision, swelling of hands and face, convulsions, fits, and high blood pressure), symptoms suggesting sepsis (e.g., high fever, foul smelling discharge, and severe lower abdominal pain). anemia, and others (unable to categorize). Too few women mentioned signs of newborn complications for analysis. This is noted and discussed in the “Discussion” section of this paper.

We used simple descriptive statistics and Fisher’s exact tests to determine if the aforementioned variables differed by women’s care-seeking status and bivariate analysis to compare care-seeking facilitator by type of complication experienced. We also conducted two multivariate logistic regression analyses to assess whether women’s attendance at two or more CMNH family meetings (both alone and with a family member) was associated with biomedical care-seeking among those who experienced a complication. In the logistic regressions, we calculated adjusted odds ratios and *p* values. Additionally, we used the SAS GENMOD procedure for generalized estimating equations to provide *p* values adjusted for clustering by *kebele*. An exchangeable correlation structure was specified. The exchangeable correlation structure was specified since women within a *kebele* are expected to be correlated due to shared community-level exposures, as well as shared health extension workers—who serve at the *kebele* level. The exchangeable correlation structure was also chosen because the ordering of observations within the cluster is arbitrary and the number of observations per cluster varies, two features which the exchangeable correlation structure allows. In the first model, control variables included the aforementioned women’s characteristics, antenatal care, and birth attendance by a skilled provider or health extension worker. In the second model, we substituted facility delivery for attendance by a skilled provider or health extension worker. Adjusted odds ratios, Wald 95% confidence intervals and *p* values were calculated for both models. Data were analyzed in SAS 9.3 (Cary, NC). Alpha was set at 0.05.

### Maternal and newborn health program inventory and health facility mapping

As part of this case study, we conducted a program inventory to identify any new maternal and newborn health initiatives operating in the former MaNHEP project area and to assess whether any elements of MaNHEP were still operating. We also conducted health facility mapping to determine the availability of trained staff, equipment, and supplies necessary to respond to maternal and newborn complications in the study area [12].

Sampling was the same for both assessments. Working with the regional health bureaus, we obtained an updated list of health facilities in former MaNHEP *woredas*. We randomly selected one health center and two health posts from each *woreda* to reduce spatial and performance bias among catchment areas of the different facilities. A district hospital, zonal hospital, and regional hospital were included for a total of 21 facilities. With the support of the regional health bureaus, a team member communicated with the facility in-charge, head, or designate. S/he described the study and the purpose of the assessments and invited their participation.

After obtaining consent using standard disclosure procedures, two teams, each comprised of one regional health bureau and one MaNHEP staff member, conducted the face-to-face interviews in a place that offered privacy. For the program inventory, they used an interview guide containing open-ended questions about the presence and characteristics of new maternal and newborn health initiatives including name, location, duration, and intervention elements. Each interview lasted, on average, 20 min. For the facility mapping, they used a structured questionnaire designed to elicit the facility type, location, hours of operation, number of trained staff, as well as the 24-h presence of trained staff, medicines, equipment, and supplies required to provide emergency obstetric and newborn care. This was coupled with a checklist to record observation of facility records, available stock, equipment, and supplies. Mapping lasted, on average, 45 min. The inventory and mapping tools were developed and standardized for use by the TRAction project country teams and are described in this supplemental issue [15].

Data were entered into Microsoft Office® Excel, de-identified, and cleaned for analysis. We conducted a simple descriptive analysis to summarize the data.

### Illness narrative

Working with the same health center and health post personnel, we identified potentially eligible cases from the catchment areas of these facilities. A case was defined as a mother and/or her newborn illness event and included the mother and witnesses to the event [12].

Potentially eligible cases were identified from birth and postnatal registers and by interviewing health extension workers, health development army coordinators, and community members. Inclusion criteria were mother resided in the study area, age 18–49 years, gave birth within the previous 6 months, willing, and able to participate. We proposed a purposive sample of 24 maternal cases (for *each woreda,* 3 cases in which the mother perceived excessive bleeding after delivery and survived and 1 case where the mother died from any complication). The final sample of consisted of 22 cases (17 cases of maternal survival, 5 cases of maternal death). Due to the challenge of finding maternal death cases, the final sample included any maternal death case whether the mother died during pregnancy before birth or after birth. Similarly, criteria for newborn cases were mother resided in the study area, age 18–49 years, gave birth in the previous 6 months, perceived her newborn became ill within 28 days of life, willing, and able to participate. We proposed 30 newborn cases (for *each woreda*, 3 cases in which the newborn survived to 28 days of life and 2 cases in which the newborn died within 28 days of life). The final sample consisted of 29 cases (16 cases in which the newborn survived and 13 cases in which the newborn died). Our inability to reach the proposed sample sizes was due to poor quality of facility records and geographic inaccessibility.

Health development army members and local community administrators helped the teams schedule appointments with potential respondents. The teams met with and screened each potential respondent for inclusion. If the respondent met the inclusion criteria, they described the study and obtained verbal informed consent using standard disclosure procedures.

The narrative method and interview guide are described in this special supplemental issue [10]. Two teams, each comprised of two experienced qualitative interviewers (one male and one female), were involved. One member elicited and audio tape-recorded the illness narrative while the other took field notes and completed the event timeline. In each case, they elicited the narrative from the mother (or family caregiver in cases involving a maternal death) but allowed the witnesses to share their views of the event. Witnesses included husbands, mothers, fathers, other relatives and neighbors. The teams worked to establish rapport with the respondents to reduce potential response bias. The narratives lasted 1.5 h on average.

Within 4 h of completing a narrative, the teams held a debriefing session in which they used a template to document their impressions of the narrative and the quality of the data and to summarize preliminary findings. Subsequently, they developed expanded field notes from memory, from the debriefing notes, and further augmented these notes with content from the recordings. Expanded notes [16, 17] capture narrative details in the voice of the respondents and include verbatim quotations and local language terms with direct translation. Responses are contextualized through interviewer comments and observations regarding the interview situation. The debriefing reports and draft expanded field notes were captured in Microsoft Office® Word and sent to the senior researcher. Data quality was enhanced through rigorous training of experienced interviewers on the study objectives and illness narrative guide; through repeated role-play and feedback; informed consent procedures; cross-checking consistency of responses through having multiple witnesses to the event; use of a timeline for clarification, recall, and verification; debriefing sessions on interview dynamics and data quality; and regular feedback on the expanded field notes for each case.

The illness narrative codebook is described in this supplement [15]. The senior local researcher trained one team member on its use in coding and developed a coding template based on the codebook in QSR International Pty., Ltd.,© Nvivo10. The senior researcher and team member coded two expanded field note reports, compared results, discussed, and aligned their coding. Subsequently, the team member coded the expanded field notes for each case and regularly submitted results to the senior researcher for review and feedback. The codebook and template were supplemented with new codes as needed during the coding.

The analysis involved multiple steps. We conducted a thematic content analysis using NVivo10. We then collapsed and re-coded care-seeking pathways into one of two categories: early biomedical (i.e., care was sought at a health facility in the first or second step of care) or late/no biomedical (care was not sought or was sought at a health facility after two steps of care-seeking). This analytic decision was based on the fact that the MaNHEP project emphasized in situation of a homebirth that mothers and family caregivers provide first aid care while arranging referral to a health facility [18]. We recognize that others may disagree with this narrow biomedical definition. Third, we entered data on socio-demographic characteristics of the mother and thematic data codes case-wise into Microsoft Office® Excel and exported these data into IBM® SPSS. We used simple descriptive analysis to describe respondent characteristics. Data on care-seeking pathways, timing, and experience are presented graphically and illustrated with case scenarios.

## Results

### Study context

The secondary analysis showed that awareness of maternal complications was low among 1027 women at baseline but substantially higher by the 2012 end line survey (Table 1) [12].

**Table 1:** Women’s awareness of pregnancy-related complications in the MaNHEP baseline (Jun-Aug 2010) and end line (May-Jul 2012) surveys, in Amhara and Oromiya regions, Ethiopia

Complication	Baseline	End line	*p* value
	(*n* = 1027)	(*n* = 1019)	
Sepsis, *n* (%)	219 (21)	598 (59)	< 0.001
Bleeding, *n* (%)	351 (34)	618 (61)	< 0.001
Pre-eclampsia or eclampsia, *n* (%)	207 (20)	534 (52)	< 0.001
Prolonged or obstructed labor, *n* (%)	116 (11)	466 (46)	< 0.001
Anemia, *n* (%)	0	141 (14)	NA
Other, *n* (%)	37 (4)	162 (16)	< 0.001

Of 1019 women surveyed at end line, 191 women reported having experienced a complication (Table 2). In order of frequency, the complications reported by women included prolonged or obstructed labor (60%), abnormal bleeding (54%), anemia (50%), pre-eclampsia or eclampsia (43%), and sepsis (35%). We expected that women who experienced symptoms of a major complication would seek biomedical care. Women who had experienced symptoms of prolonged obstructed labor were significantly more likely to have sought biomedical care compared with women who sought care for other conditions (60% versus 40%, respectively, *p* = 0.02). Surprisingly, women who experienced symptoms of sepsis were significantly less likely to have sought biomedical care compared those who sought care for other conditions (35 versus 52%, respectively, *p* = 0.02). Although care-seeking for abnormal bleeding was a topic in the MaNHEP family meetings, women who reported this condition were not more likely to have sought care compared with women who sought care for other conditions (54 versus 40%, respectively, *p* = 0.21).

**Table 2:** Women’s experience of pregnancy-related complications by biomedical care-seeking in the MaNHEP end line (May-Jul 2012) survey in Amhara and Oromiya regions, Ethiopia

Complication		Sought biomedical care		*p* value
		Yes (*n* = 85)	No (*n* = 106)	
	*n*	*n* (%)	*n* (%)	
Sepsis	86	30 (35)	56 (65)	0.02
Abnormal bleeding	39	21 (54)	18 (46)	0.21
Pre-eclampsia or eclampsia	80	34 (43)	46 (57)	0.66
Prolonged or obstructed labor	43	26 (60)	17 (40)	0.02
Anemia	16	8 (50)	8 (50)	0.79
Other	36	17 (47)	19 (53)	0.85

The first regression model (Table 3) shows that women who sought biomedical care for one or more pregnancy-related complications were significantly more likely to have attended CMNH family meetings and to have given birth in a health facility. The second model (Table 4) shows they were more likely to be from a family without land and to have given birth with a skilled provider or health extension worker. Nurses facilitated one-third of all referrals; families, traditional birth attendants, and health extension workers were also involved (data not shown) [12].

**Table 3:** Characteristics of women most associated with biomedical care-seeking among 191 women who experienced one or more pregnancy-related complications (model 1)

Characteristics	OR (95% CI)	*p* value
Amhara region of residence (vs. Oromiya)	2.02 (0.91, 4.50)	0.08
Age, years	1.07 (1.00, 1.14)	0.06
Educational level, highest completed		0.27
Any primary (vs. none)	0.61 (0.24, 1.57)	–
Any secondary or higher (vs. none)	3.00 (0.36, 25.36)	–
Husband has any education (vs. none)^a^	1.80 (0.81, 3.99)	0.15
Any land ownership (vs. none)	0.47 (0.18, 1.22)	0.12
Any ANC from skilled provider or HEW	1.02 (0.42, 2.48)	0.97
CMNH Family Meeting attendance^b^		0.04
≥ 2 meetings, alone (vs. < 2)	2.36 (0.81, 6.91)	–
≥ 2 meetings, with family team (vs. < 2)	0.59 (0.25, 1.37)	–
Gave birth in any health facility (vs. home or other)	8.77 (3.59, 21.43)	< 0.001

^a^Not currently married (5)

^b^Missing (19)

**Table 4:** Characteristics of women most associated with biomedical care-seeking among 191 women who experienced one or more pregnancy-related complications (model 2)

Characteristics	OR (95% CI)	*p* value
Amhara region of residence (vs. Oromiya)	1.40 (0.69, 2.86)	0.35
Age, years	1.05 (0.99, 1.11)	0.12
Educational level, highest completed		0.40
Any primary (vs. none)	0.79 (0.33, 1.88)	–
Any secondary or higher (vs. none)	2.96 (0.42, 20.65)	–
Husband has any education (vs. none)^a^	1.79 (0.85, 3.74)	0.12
Any land ownership (vs. none)	0.39 (0.16, 0.95)	0.04
Any ANC from skilled provider or HEW	1.02 (0.42, 2.48)	0.97
CMNH Family Meeting attendance^b^		0.06
≥ 2 meetings, alone (vs. < 2)	2.08 (0.75, 5.74)	–
≥ 2 meetings, with family team (vs. < 2)	0.60 (0.27, 1.32)	–
Gave birth with a skilled provider or HEW (vs. unskilled or alone)	2.74 (1.36, 5.53)	0.005

^a^ Not currently married (5). ^b^ Missing (19)

The 2014 program inventory showed that the Ministry of Health had enhanced maternal and newborn health services by introducing new initiatives such as routine performance review, *woreda* ambulance services, increased posting of midwives in health centers, monthly pregnant women’s conference focusing on birth preparedness and complication readiness conducted at health centers, the government’s home free delivery policy that emphasizes health facility delivery and making health facilities “family-friendly,” and maternal death surveillance and response. These initiatives were introduced nationwide including in the former MaNHEP area. It also showed that aspects of the MaNHEP interventions had been integrated into the health services. For example, community health volunteers were absorbed into the health development army, many in leadership roles, best practices were adopted for identifying and registering pregnant women for antenatal care and facilitating postnatal care follow-up by health extension workers, and educational materials were absorbed into the pregnant women’s conference and health development army activities. Lastly, the Ministry of Health continued to support the training of doctors, nurses, and midwives in emergency obstetric and newborn care and equip facilities to provide these services [12].

Facility mapping showed that the secondary and tertiary hospitals were able to perform the functions of comprehensive emergency obstetric and neonatal care, while four of six health centers were able to perform the functions of basic emergency obstetric and neonatal care; moreover, most facilities had done so within the last 6 months (Table 5). All essential medications except for misoprostol were available, and there were no stock-outs in the last 6 months. Misoprostol was available in only four facilities [12].

**Table 5:** Emergency obstetric and newborn care availability and performance

Signal function	Available today		Performed last 6 months	
	Hospital (*n* = 3)	Health center (*n* = 6)	Hospital (*n* = 3)	Health center (*n* = 6)
Parenteral antibiotics	3	6	3	6
Uterotonics	3	6	3	6
Parenteral anticonvulsants	3	5	3	4
Manual removal of placenta	3	6	3	6
Removal of retained products	3	6	3	6
Assisted vaginal delivery	3	5	3	5
Neonatal resuscitation	3	6	3	6
Blood bank/blood transfusion	2	0	2	0
Cesarean section	3	0	3	0

### Illness narrative sample characteristics

Mothers in the maternal cases were 31.8 ± 9.6 years of age (range 20–52 years). A majority (12/22) had no formal schooling. Of mothers who had attended school, the highest grade completed was grade 8. Of 17 surviving mothers, 11 gave birth at home and the remaining six gave birth in a health facility or on the way to a health facility. Of five deceased mothers, four died during pregnancy while one died after birth; all in a health facility.

Mothers of the newborn cases were 28.2 ± 6.2 years of age (range 19–42 years), and a majority also had no formal education (18/29). Of those who had attended school, the highest grade completed was grade 10. Of the 16 surviving newborns, five were born at home, 11 were born in or on the way to a health facility. Of the 13 deceased newborns, all but one died within the first week of life and nine died at home.

### Care-seeking pathways, timing, and experiences

#### Maternal cases

**Fig. 1:**
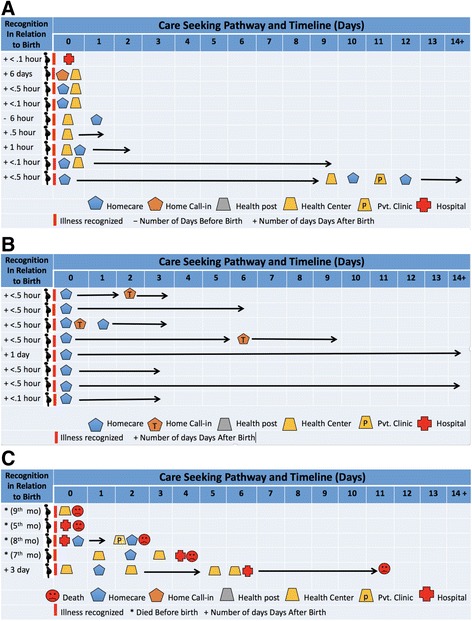
Biomedical care-seeking pathways and timing for maternal cases

Care-seeking pathways and timing are depicted in Fig. 1a–c. The day of illness recognition, indicated by the small red bar, is set at day 0. Reading from the left to right, the timeline is indicated in days from the day of illness recognition. The far-left column shows the number of days before (**−**) or after birth (**+**) that the illness was recognized. The arrow (→) indicates continuing symptoms. Icons described in the legend show the sequence of care-seeking behaviors (e.g., home care = blue house, calling a traditional provider or formal health care provider into the home to provide advice or care = orange house, health post care = gray trapezoid, health center care = orange trapezoid, private clinic care = orange trapezoid with P, and hospital care = red cross). Figure 1a–c show that mothers and families pursued different and sometimes multi-step strategies. Nine families of the surviving 17 mothers sought early biomedical care (Fig. 1a). Of these nine mothers, one sought care only from a health center, another only from a hospital, and the remaining mothers took two or more care steps including care at home and at a health center or private clinic. Factors facilitating care-seeking were advice and counseling by a traditional birth attendant or health extension worker to seek care for excessive bleeding, including during antenatal care, motivated them to seek care. Some of the mothers had had prior experience with bleeding. The estimated time from illness recognition to decision to seek care ranged from immediately to within 1 h in all but one case. Families thought the mother’s symptoms were serious in all but one case.

The following scenario, told by the mother, her mother-in-law and sister-in-law who were witnesses to the illness event, gives a sense of factors involved in care-seeking decisions such as perceived severity of the situation, immediate use of traditional home care, and environmental and logistical constraints often involved when seeking a higher level of care.

*I planned to give birth at home, as I had done with my other babies. My labor pains started around 3 pm and gradually increased in strength. About 6 pm, I sent someone to call my mother-in-law and sister-in-law who were attending a social gathering. Around 2 am, the baby was born, but the placenta did not come. Blood started to flow and was so heavy that I grew weak, drowsy and started vomiting. The mother-in-law and sister-in-law said they had planned to take L to the health center the next morning. But, around 2L30 am, seeing they could not control the bleeding and that the situation was getting worse, they and L’s husband decided to call the health extension worker to bring an ambulance. Meanwhile, thinking the bleeding might be due teyazua--a spirit that comes to take a life when a person is angry-- they roasted coffee and put perfume on L’s head and clothing. But she continued to bleed.*

*The ambulance was unable come all the way to the house because it was dark, raining and the road was muddy. We called neighbors to help carry L, lying in her bed, to the ambulance parked at a nearby a bridge. It was difficult going and took over an hour. Meanwhile, she continued to bleed. According to L, it was like the sea, without limit, it clotted and came out… falling like a grinding stone. I felt like I was giving birth again. They said it was only blood but I was thinking it was a twin birth. I was exhausted, felt cold and finally lost consciousness. A cousin met us at the bridge and went with us to the health center. My mother-in-law stayed behind with the baby.*

*According to sister-in-law, we arrived at the health center around at 4 am. The health workers hesitated to treat L because we had left the baby at home. They ordered us to go back for the baby. So I went home to get the baby. L was finally admitted. They removed the placenta and gave her a bag of saline and an injection. L regained consciousness around 6 am. The bleeding had finally slowed, though she said that she still felt cold. She was discharged around 1 pm for home.*

The remaining eight families did not seek care or sought non-biomedical assistance, although in three cases, the mother’s sister or husband went to obtain medicines for the mother from a traditional birth attendant or pharmacy (Fig. 1b). Six families thought the mother’s symptoms were serious. Factors impeding early care-seeking from biomedical providers were rain and muddy roads, nighttime, distance, lack of transportation or money, and the mother’s condition (e.g., too weak or unclean and traditional postpartum restrictions on movement).

The following story gives a sense of nearly insurmountable social, financial, and logistical barriers to care faced by a poor, single mother.

*The pains started when the cock crowed the first time around 3:00 am. I called the neighbors repeatedly, but no one came. When the baby came out the cock crowed for second time around 5:30am. Then I fainted. When I became conscious, I shouted loudly for someone to help me, again and again, but no one came. The baby was lying on the ground so I wrapped and held him while I tried to find the kerosene lamp, a razor blade and some thread. I tied and cut the cord, separating the baby. The bleeding was too much before and after I separated the baby. I walked around the house to help push the placenta out. It came after a while. I tried to breastfeed the baby but he couldn’t suck since I didn’t have milk yet.*

*No one wanted to help me because I got pregnant by my relative and they thought I did this deliberately. The reality was different. One day the baby’s father came to visit as usual and wanted to stay longer. I told him to go back to his home because it was dark and our sleeping time, but he refused. Suddenly he grabbed and raped me, and then disappeared. His family knew what was going on since their house is close to my house. They didn’t want to help… they wanted me to die. My parents are not alive but I have a brother and sisters who also have rejected me. I live alone with my 4-year old boy without support.*

*I have had fever at night and bleeding has continued, though the amount has decreased in the last 7 days. I have used all of the clothes I have to handle the bleeding, but it is too much. I changed clothes 3-4 times; everything is completely soaked, drowned with blood and big clots like a fist. My clothes are still not washed [The interviewers were able to see that the mother’s clothing was completely soaked with dried blood and even their seats had dried blood on them]. I thought I was going to die. I haven’t had treatment, not even herbs, though I tried to send my boy to bring some tunjit and fanos. He said no because he is too young. After a few days, one neighbors ground some grain and handed me the flour through the door. She would not come in. I can’t go out since I delivered recently and do not have my strength back yet. I can’t go to the health facility because I have no one to escort me. Also I have financial and transport problem to go to there. I tell you I am not supposed to survive. It is God who has saved my life and has kept me alive so far.*

Families of mothers who died sought care from biomedical providers (Fig. 1c)*.* One went directly and only to a health center and another directly and only to a hospital while the remaining families took from 4 to 5 care steps, shuttling between home, health center, private clinic, and/or hospital. Perceived severity of symptoms varied. One family thought symptoms were serious, another not serious, while the remaining families initially thought symptoms were not serious, but later serious. Four mothers died undelivered; none died from excessive bleeding. Symptoms common in three cases were severe headache, vomiting, and fever. With one exception, families of mothers who died sought care from the day illness was recognized. The estimated time from recognition to decision to seek care ranged from immediately to within 16 h.

This story, told by a husband who witnessed his wife’s illness and death, illustrates a desperate situation of repeated, unnecessary delays, an apparently inexperienced provider, and disrespectful, poor quality care.

*A was 7 months pregnant. She had started coughing on Thursday afternoon. The next day, I went to the farm. When I came back home, around 4 pm, I noticed that her cough was much stronger and it had an odd sound. She was having difficulty breathing because the coughing was so constant and she said it was painful.*

*That night, around 2 am, I called my relatives to come help me take her to the health center. We could not carry her on her bed because she couldn’t breathe lying down. So, we took her sitting up in a chair. We got to the health center before sunrise. A guard told us to wait for the doctor to come [He was not in the compound.]. He came after an hour. The doctor was young. He examined my wife and told us that she was fine. He then gave her an injection and some tablets and told us to take her back home.*

*Instead, we took her to my brother’s house, where we stayed the whole day. We gave her the tablets… but she didn’t get better. Later that same day I called the doctor to tell him that she was getting worse. He again said that she was fine, not to worry. He said to bring her to the health center on Monday and he would examine her. What can I do if a health professional says she is fine? So, I waited until Monday to go back to the health center. The doctor [same] said that it is a Muslim holiday and they are not working. He also said that is no electricity [for lights] to examine her. I asked him to please refer us to another health facility and he said that I am not the one who decides this. It is he that decides these things. I begged him in the name of Christ… why won’t he help my wife as he can see that she is having difficulty breathing? I asked him again to give us a referral paper, but he refused. He instead started giving her saline drip and left the room.*

*By this time A’s face had swelled and, after she finished the saline, she lost consciousness. I screamed. He came back and checked her and, again, said she is fine. Alma spent the whole night like this. The next morning, the doctor referred her to the hospital and called an ambulance. Alma passed away soon after we arrive at the emergency room. There they insulted me, asking, “Why do you bring us a dead body?” I told them that I had taken her to the health center and that the doctor had refused to give me a referral paper. They were very angry and said that I should sue them. I was so sad…*

*I rented a car and brought her body home. Since that day I see health professionals who work in this health center as my enemies. If it [my wife’s illness] was above their capacity, they could have referred her to hospital. Instead, they kept her from Saturday to Tuesday morning without doing anything. I couldn’t do anything… I had enough money in hand, but I couldn’t do anything… she died of negligence.*

#### Newborn cases

**Fig. 2:**
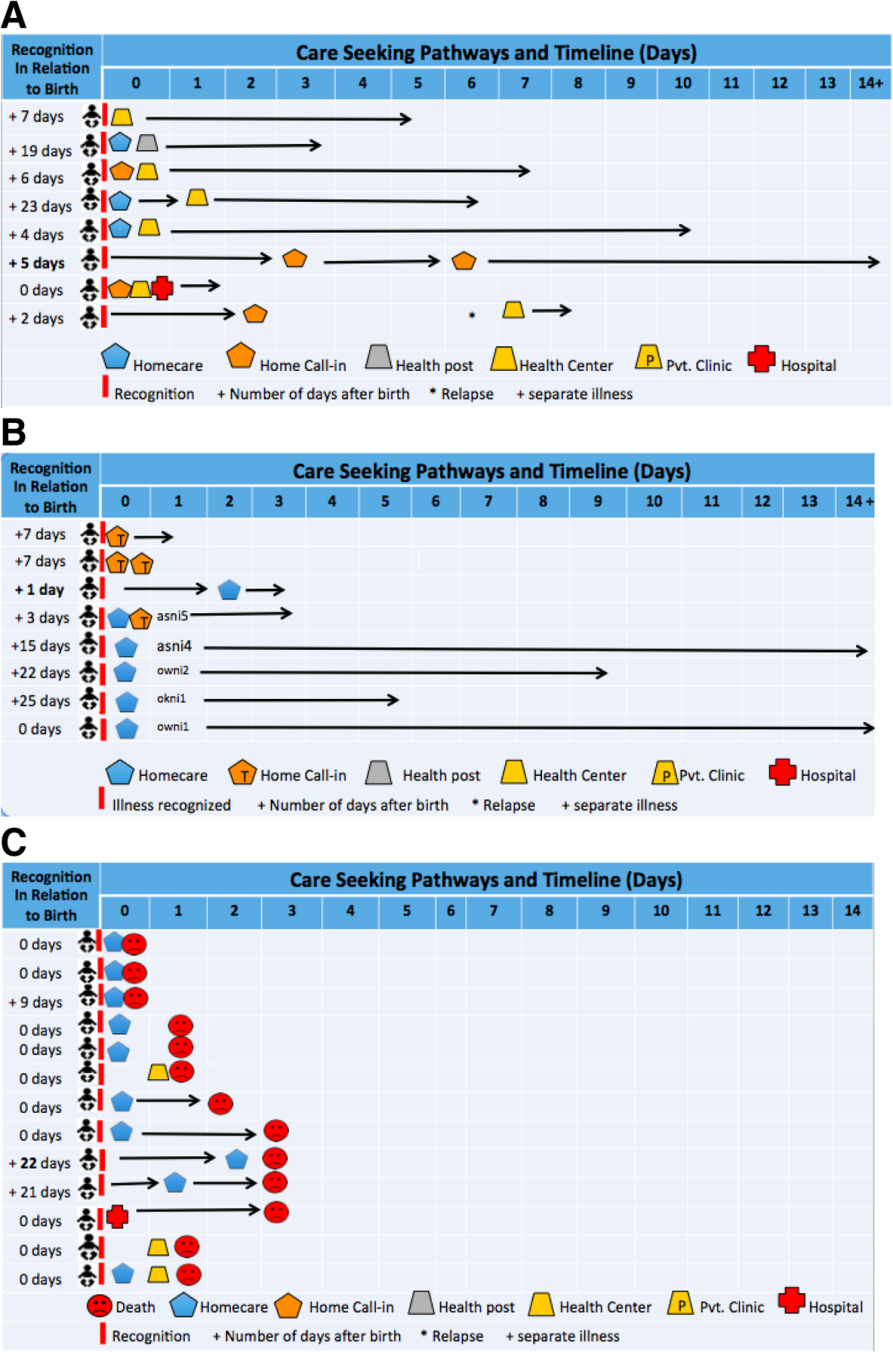
Biomedical care-seeking pathways and timing for newborn cases

Eight families of surviving newborns sought care from biomedical providers (Fig. 2a). One family went directly to a health center, and the remaining took two or more care steps. Six thought the baby’s symptoms were serious. Factors facilitating care-seeking included advice of health extension workers, the ability of health extension workers to refer, free care, and fear of traditional care. Care-seeking from the time of recognition occurred within 1 day in five of the eight cases.

This story illustrates a family that acted on the advice of their health extension worker.

*After the baby was born, we noticed that he was not well. This was around 4 am. He was not suckling very well and vomited afterwards trying. He was sneezing repeatedly and seemed to be uncomfortable. We thought it may be due to the cold weather or maybe a common cold and we did not think it was serious. I didn’t do anything for him except keep trying to suckle. By 10:00 am, he was much weaker and was unable to suckle. This was becoming serious.*

*My husband called the health extension worker to try and get treatment from her. She came around 11:00 am. When she felt the baby’s forehead, she immediately said we should take the baby to the health center. We left for the health center, reaching there at around 11:40 am. The moment we reached there, the health worker examined my baby. She said that his illness was due to a cold and prescribed an injection and syrup for 7 days. After this, we went home. I gave the syrup and by 8:00 pm I saw some improvement-- he started to breastfeed. By the 7* ^*th*^ *day he was completely well. I am very happy about the treatment and service that he received because he got his health back in a very short period of time.*

The remaining eight families of surviving newborns sought no care or non-biomedical assistance. Five of these families provided home care only, while others called in a traditional birth attendant or traditional healer for treatment (Fig. 2b). All thought their newborn’s symptoms were serious. Yet, factors impeding care-seeking from a biomedical provider included rain, nighttime, distance, lack of transportation or money, the mother’s condition (e.g., too weak and/or unclean and traditional postpartum restrictions on movement) as well as the baby’s condition (e.g., too small or too young, not baptized, and the illness requires a local treatment).

This story, told by the mother, illustrates a situation in which the family attributed the baby’s symptoms to *fallen uvula*, leading to their seeking a local healer for assistance––despite some disagreement as to best course of action. *Fallen uvula* was reported in half of the cases of inappropriate care-seeking and is described in the “Discussion” section.

*I gave birth to a healthy baby boy in the health center on August 21. On the baby’s 7th day of life, at 6 am, I observed that the baby had a fever, hiccups and was unable to suckle. His situation became severe within a few hours. I sent someone to call my mother to come check the baby and bring enzirt and cotton. When my mother arrived, she checked the baby and found that the glottis (uvula) had dropped… it was red and swollen. We discussed this with some neighbors and decided the glottis should be cut. We agreed that this problem is natural to newborn babies and only by cutting the glottis can the baby be cured permanently.*

*My husband and a neighbor disagreed, suggesting we take the baby to the health center. But we were worried because recently, a baby with a glottis problem had died in our village. The family didn’t take the baby to a health facility and also didn’t have the baby’s glottis cut. I also felt that my baby was too small to be taken to the health center, I was still in seclusion and, besides, the health center was too far away. People do not fully believe that medicine given in the health center can cure a glottis problem without some proof. This will take some convincing. When that happens, they will go.*

*In the end, my mother went to call a famous healer to the house. When she got to his farm the man was harvesting barley. He asked why we didn’t bring the baby to him, and when the mother told him that they couldn’t bring the baby, he dropped his sickle and came. They arrived around 8:30am. The man brought a special tool for cutting the glottis, one that had been passed down from his father. He boiled the tool for half an hour, then cut the baby’s glottis and boiled the tool again. He put the cut glottis on the soft place on baby’s head. My husband paid the man 20 birr.*

*The baby was not feeling better the whole day after the glottis was cut. He was crying constantly, still had difficulty suckling, and a fever. He was like this all through the night. However, he fully recovered the following evening. I was very relieved and happy. After a week, I felt sure that my baby was out of danger.*

Four families of the 13 deceased newborns went directly to a health center or hospital. All thought their newborn’s symptoms were serious. Factors facilitating care-seeking were the advice of the health extension worker, the health extension worker’s ability to refer, and proximity of the health facility. The estimated time from recognition to decision to seek care ranged from one to 22 h.

Nine families, however, did not seek early biomedical care. Six thought their newborn’s symptoms were serious (Fig. 2c). Factors impeding care-seeking were similar to those mentioned by the families of babies who survived. In addition, they mentioned difficulty calling for an ambulance, losing hope, belief that the baby would get better, and fear of *evil eye*.

The following story was told by a mother who witnessed her newborn’s illness and death.

*In the 7th month of my pregnancy, I felt labor pains after sunset but I refused to go to the health center for delivery because I had had a miscarriage at a hospital before. After experiencing bleeding later that night, I decided to go the health center. We reached the health center at about 5 am but I was urgently referred to the hospital and arrived there in an ambulance by 10 am. They examined me and waited for me give birth naturally. But they soon decided to have me deliver with an operation. I gave birth to twin girls. They brought them to me on the second day. They advised me to breastfeed my babies and told me it would help to massage my breasts because I did not have milk yet. Both babies were able to suckle but they got milk only on the following day.*

*We returned home that day and they both were breastfeeding well, but they both felt cold to me. I wrapped them in warm clothes and they slept a lot. One was especially cold and did not breathe or breastfeed well. I was worried about her and was afraid that she might die.*

*At the end of the 3* ^*rd*^ *week, on a Friday, she was unable to suckle or to open her eyes. She slept continuously. The next day, I checked her uvula and saw that it had fallen. To treat this, I chewed seven kernels of barley and placed them on the soft spot on her head [fontanelle]. I repeated this in the afternoon to help shrink the uvula. My husband suggested taking her to the health center but I did not agree because they did not have an incubator and she was too young and weak to take syrup or an injection. He also wanted to take her to the hospital but my mother discouraged him from taking a hopeless baby there.*

*The baby was able to suckle some on Sunday and Monday. Late, on Monday, she was too weak and unable to breastfeed. She became colder and her breathing changed. I just hugged her and watched her condition, until she stopped breathing and died. She was very small but she was so beautiful, her hair, her eyes and her nose were beautiful. God made her perfectly.*

#### Differences in care-seeking by type of case

Including all cases, timely biomedical care-seeking occurred more often for mothers than for newborns (14/22 or 64% for mothers and 11/29 or 38% for newborns, respectively). Considering only the cases culminating in a death, appropriate care-seeking occurred substantially more often for mothers than for newborns (5/5 or 100% versus 4/13 or 31%).

Excluding cases in which a mother died undelivered, 20/47 or 43% of mothers gave birth at home while 27/47 or 57% gave birth on the way to or in a health facility. Considering these maternal and newborn cases separately, timely biomedical care-seeking did not appear to be related with the place of birth in either maternal or newborn cases.

## Discussion

The MaNHEP baseline and end line surveys showed that between 2010 and 2012, facility births increased significantly in the Amhara but not in Oromia study area, although the percentage births taking place in a health facility for both regions combined at end line was low—only 15% [4]. This case study in the former MaNHEP project area 2 years later shows that 6 of 17 (35%) mothers who survived a major complication of pregnancy gave birth in a health facility. While the survey and case study samples are not comparable, this finding is encouraging. This proportion is somewhat higher than that described in the Ethiopia Demographic and Health Survey 2016 Key Indicator Report for Amhara and Oromia regions (27 and 19%, respectively, reflecting the situation 5 years preceding the survey) [19].

Although there were improvements in women’s awareness of maternal complications by the end of the MaNHEP project (2012 end line survey), only prolonged obstructed labor was significantly associated with biomedical care-seeking. Excessive bleeding, an important focus of the MaNHEP CMNH family meetings [18], was not significantly associated with biomedical care-seeking, although just over half of such women sought care for perceived excessive bleeding (54%) [19]. In reading the case study narratives conducted in 2014, some descriptions of the characteristics of bleeding experienced suggest clinically normal blood loss and lochial discharge after birth, though perceived to be excessive by women and witnesses.

That women in the MaNHEP baseline and end line surveys rarely spontaneously mentioned newborn complications [12–14] is possibly reflected in the difference in maternal and newborn care-seeking observed in cases in which the mothers and newborns died (100 versus 31%, respectively). The families of four of five mothers who died sought care for illness occurring during pregnancy and died undelivered in a health facility, while the last mother gave birth and died in a health facility. Symptoms these mothers experienced that triggered care-seeking were severe headache, vomiting, fever, cough, lack of fetal movement, etc. Perceived severity of these symptoms varied. Why only four of 13 families sought biomedical care in the case of newborns who died is not entirely clear. Symptoms mentioned by nine families who did not seek care included difficulty breathing, poor feeding, weakness, and cold body (to touch). Three of these nine newborns were understood by the parents to have been born premature. From a biomedical perspective, these symptoms would be considered serious and should have triggered timely biomedical care-seeking [19]. One possible explanation is perceived severity of the newborns’ symptoms. The families who sought biomedical care perceived the babies’ symptoms to be serious or hopeless. The families who did not seek such care perceived symptoms variously (e.g., serious or hopeless, serious but would get better, and initially not serious, but later serious). This suggests some uncertainty about the meaning of symptoms and expected health outcome or prognosis, which may have delayed a decision to seek care or prompted a decision not to seek care. Other barriers explicitly mentioned by these nine families included logistical barriers such as distance, lack of a cell phone to call the health extension worker for an ambulance, lack of money for transport, the fact that it was nighttime, as well as concern that the baby was too small or too weak and/or that the baby might be exposed to *evil eye*. Families who did not seek biomedical care in cases where the ill newborns survived, as well as where the ill mothers survived, also mentioned these same barriers, among others (e.g., the appropriate response was traditional care). Other researchers have observed that while there are large variations, care-seeking for newborn illnesses appears to be low in general and highly contextual and remains a key challenge to improving neonatal mortality [20].

Considering all cases, most mothers and families recognized and responded to symptoms in a timely manner. Perceived severity and place of birth do not appear to be associated with timely biomedical care-seeking. Factors facilitating care-seeking were similar across the cases and include advice and counseling by trained community volunteers and health extension workers, proximity of health extension workers who can refer to a higher level of care, free care and, in some cases, perceived ineffectiveness of traditional care. Factors impeding care-seeking, many of which are described above, were time-of-day, bad weather, poor road conditions, distance, poor communications, lack of transportation or money, the mother’s condition (e.g., too weak and or unclean and traditional postpartum restrictions on movement outside of the home), the baby’s condition (e.g., too small, too weak or too young, and not yet baptized), and, in some cases, fear (e.g., of *mitch*, a metaphysical condition associated with exposure to the elements or *evil eye*, a gaze or stare superstitiously believed to cause material harm) [21]. Other researchers have identified similar factors [22–31]. Somewhat unique in this case study is *fallen uvula*, a folk illness that mothers and families associate with poor feeding, vomiting, fever, and a red swollen uvula. The illness is considered life threatening if the uvula ruptures, and the preferred treatment is uvulectomy performed by a traditional healer [32]. Although uvulectomy is decreasing, it is still widely practiced in Ethiopia [7].

A descriptive case study employing mixed-methods was the appropriate approach for answering the questions posed in this research. Compared with other methods, it permits a better understanding of the changing patterns of care-seeking over time, in relation to events. Yet, the approach is challenging to use. Challenges include selecting the guiding framework and relevant data sets to address the questions, as well as development of and rigorous adherence to different types of data collection and analytic procedures. Most of these challenges were addressed through a series of workshops hosted and led by the USAID/URC team. The workshops included proposal development, training in illness narrative data collection methods and procedures, instrument standardization and pretesting, analysis, and writing. Illness narrative incorporated multiple means for assuring data quality such as the inclusion of an illness event timeline and iterative neutral probes when eliciting the narrative to stimulate recall of the event, triangulating the memories of the event by the mothers and other witnesses, and regular review and feedback to interviewers by the senior local researcher about the nature and quality of the narrative data. Whether the method would elicit the same narrative if repeated is unknown. Moreover, the method requires adequate interviewer training in elicitation techniques and support as the narratives often evoke painful memories.

## Conclusion

We hypothesize that improvements observed in women’s use of a health facility for birth, awareness of maternal complications, and referral for some experienced maternal complications during the MaNHEP project [4] continued as a result of subsequent government priorities and new initiatives in the former project area. These initiatives employed a variety of demand- and supply-side interventions aimed at reducing maternal and newborn morbidity and mortality [10, 31]. We find it remarkable that biomedical care-seeking for maternal complications increased despite sometimes disrespectful, poor quality care*—*a known barrier [32–34]. It is concerning that biomedical care-seeking for newborn complications including prematurity has not increased to the same extent. There is a need for ongoing concerted efforts to (1) reduce geographical, logistical, and financial barriers to care-seeking; (2) improve the acceptability and quality of newborn care in health posts and health centers, as well as basic and comprehensive emergency obstetric and newborn care in health centers and hospitals, respectively; and (3) contextualize community behavior change communications and provider counseling. For example, messaging would be more effective if local beliefs about newborn illnesses such as *fallen uvula*, perceived maternal and newborn vulnerability, and related postpartum restrictions on maternal (and newborn) movement outside of the home, and outcome expectations are incorporated and addressed as these clearly influence care-seeking. The Ethiopia case study findings suggest that if the apparently effective government initiatives continue and the aforementioned needs are addressed, then illness recognition and appropriate care-seeking will further improve.

## Abbreviations

FMoH: Federal Ministry of Health
HDA: Health Development Army
HEW: Health extension worker
MaNHEP: Maternal and Newborn Health in Ethiopia Partnership
RHB: Regional Health Bureau
URC: University Research Company, LLC
USAID: United States Agency for International Development
